# Comparison of ultrasonographic versus infrared pupillary assessment

**DOI:** 10.1186/s13089-020-00188-1

**Published:** 2020-08-14

**Authors:** Christian D. Yic, Gabriel Prada, Sergio I. Paz, Leandro Moraes, Julio C. Pontet, Marcos E. Lasso, Alberto Biestro

**Affiliations:** 1 Department of Critical Care Medicine, Hospital de Clínicas, Montevideo, Uruguay; 2 Department of Critical Care Medicine, Hospital Pasteur, Montevideo, Uruguay; 3 Department of Critical Care Medicine, Mayo Clinic, Jacksonville, USA

**Keywords:** Ocular ultrasonography, Ocular ultrasound, Pupillometry, Pupillary assessment, Traumatic brain injury, Brain herniation

## Abstract

**Objectives:**

To evaluate the correlation between ultrasonographic and infrared pupillary assessments in critically ill patients, including neurocritically ill patients.

**Design:**

Prospective, observational study.

**Setting:**

Tertiary teaching hospital intensive care unit (ICU) in Montevideo, Uruguay.

**Patients:**

Twenty-six adults patients with age 18 or older admitted to the intensive care unit with and without neurologic pathology. A total of 212 pupillary measures were made between ultrasonographic pupillary assessment (UPA) and infrared pupillary assessment (IPA).

**Interventions:**

This was a study that utilized non-invasive (minimal risk) ultrasonographic and infrared pupillary assessment in patients admitted to the ICU. Time between UPA and IPA in a single patient was consistently less than 3 min.

**Measurements and main results:**

There was a strong positive association between UPA and IPA (right eye [OD]: *r* = de 0.926, *p*-value < 0.001; left eye [OS], *r* = 0.965, *p*-value < 0.001), also observed in the group of neurocritically ill patients (OD: *r* = 0.935, *p*-value < 0.001; OS: *r* = de 0.965, *p*-value < 0.001). Taking IPA as reference measure, the percent error for all subjects was 2.77% and 2.15% for OD and OS, respectively, and for neurocritically ill patients it was 3.21% and 2.44% for OD and OS, respectively.

**Conclusions:**

Ultrasonographic pupillary assessment is strongly correlated with infrared pupillary assessment in critically ill patients, including neurocritically ill patients. Ultrasonographic pupillary assessment is a quick, feasible, non-invasive method that allows accurate pupillary assessment, particularly neurologic function, in patients in whom a more precise measurement of the pupil is required or eye opening is not possible (e.g., periorbital edema due to traumatic brain injury).

## Introduction

Pupillary assessment (i.e., bilateral evaluation of pupils size, shape, symmetry, and reactivity) is a cornerstone of the neurologic physical examination in the critically ill, particularly the neurocritically ill. Systematic pupillary assessments are routinely performed in the critically ill because they can render early signs of neurologic deterioration, which, in some cases, may be the only clinically obtainable sign. The Brain Trauma Foundation’s guidelines for the management of severe traumatic brain injury acknowledge the evaluation of pupils’ size and reactivity to light as source for early prognostic signs of neurologic pathology [1].

There are reports of pupillary assessment as far back as 1929, when Otto Lowestein first developed a technique based on the analysis of simple, direct, visual evaluations [2]. Nonetheless, despite remarkable technologic advances and substantial improvements on the understanding of the central nervous system, the pupillary examination has not much changed during the last century. Regarding the conventional, visual pupillary assessment, there is considerable intra- and inter-observer variability due to inconsistency on several factors, such as illumination of patient’s room, examiner’s visual acuity and experience, and intensity and technique of light stimuli. Therefore, alternative techniques have been proposed [3, 4].

The most accepted alternative technique is the infrared pupillary assessment (IPA), first described in 1958 by Lowestein himself, and then, as from 1993, extensively studied by Merlin Larson; by these means, both of Lowestein and Larson ended up elucidating the association between pupillary abnormalities and brainstem injuries or pharmacologic effects [5, 6]. In 2003, Taylor et al., using an infrared pupillometer, established an association between intracranial pressure and pupillary abnormalities in patients with acute brain injury [7]. In 2011, Chen et al. using the infrared pupillometer, described a significant inverse relationship between decreasing pupil reactivity and increasing intracranial pressure; the first evidence of pupil abnormalities occurred, on average, 15.9 h prior to the time of the peak of intracranial pressure [8]. Today, the IPA is being increasingly adopted as a routine part of the neurologic examination, supported by a growing body of literature demonstrating its reliability, accuracy, and ease of use. Automated pupillometry allows rapid, non-invasive, reliable, and quantifiable assessment of pupillary function which may allow rapid diagnosis of intracranial pathology that affects clinical decision-making [9, 10].

On the other hand, the first report of ocular ultrasound was made in 1956 [11]. Technologic advances in ultrasound devices have since allowed implementing ocular ultrasound in the evaluation of several ophthalmologic pathologies such as ocular trauma and intraocular foreign body identification [12–14]. However, the ultrasonographic evaluation of the pupillary diameter and pupillary light reflex has not been well studied, let alone implemented. In cases when direct visualization of the pupil is not possible due to soft tissue injury that precludes eye opening, alternative techniques such as IPA and LED-based perimetric pupillometry have been proposed; unfortunately, most of these techniques do not overcome the physical barrier placed by the soft tissue, rely on advanced devices that are not generally available in emergency situations, and require of specialized technical support [15–17]. In this context, UPA is particularly useful because it offers a simple yet accurate alternative that can be performed with small, portable devices at the point of care.

To the authors’ knowledge, few reports have been published regarding UPA application and clinical relevance, mostly in emergency medicine and critical care medicine. Nevertheless, none of them have evaluated and compared UPA’s accuracy to standard techniques such as IPA [18–21].

### Objective

To evaluate the correlation between ultrasonographic and infrared pupillary assessments in critically ill patients, including neurocritically ill patients.

## Methods

### Study design and study population

This was a prospective, observational study following the Helsinki Declaration and approved by the institutional review board. Patients older than 18 years who were admitted to the ICU of the Hospital de Clínicas, Montevideo, Uruguay, from October 1, 2018 to March 1, 2019 were included. Exclusion criteria were patients with ophthalmologic or periorbital pathology, including abnormal pupillary anatomy or neurologic assessment at baseline, or with facial or periorbital soft tissue edema that precluded eye opening, and therefore adequate IPA.

Upon admission to the ICU, consent was obtained from patients’ family members. Disease severity was determined following the Glasgow Coma Scale, and electronic medical records were reviewed to obtain demographics (age, gender, race), vital signs (heart rate, respiratory rate, blood pressure, temperature), and hemodynamic parameters (if any).

Non-invasive (minimal risk) ultrasonographic and infrared pupillary assessments were performed during same visit to the patient. Lights in patients’ room were turned off and windows and curtains were closed, thereby ensuring homogeny in rooms’ illumination regardless of time of the day.

Because UPA required manual measurement of pupillary diameter, we performed UPA first and then IPA, thereby reducing potential source of observer bias. The time frame between UPA and IPA was less than 3 min, during which there was no administration of new medications or physical maneuvers or activities on the patient. The examinations, both UPA and IPA, were performed at any time during ICU stay, by three authors (C.Y, S.P and M.L) who were experienced in UPA and IPA. On some patients, two or more pupillary examinations (UPA and IPA) were performed, each on different days while still in the ICU. The ultrasound and infrared devices and the information they yielded were used as standard of care; however, the devices themselves were not investigated.

### Ultrasonographic pupillary assessment (UPA)

**Fig. 1:**
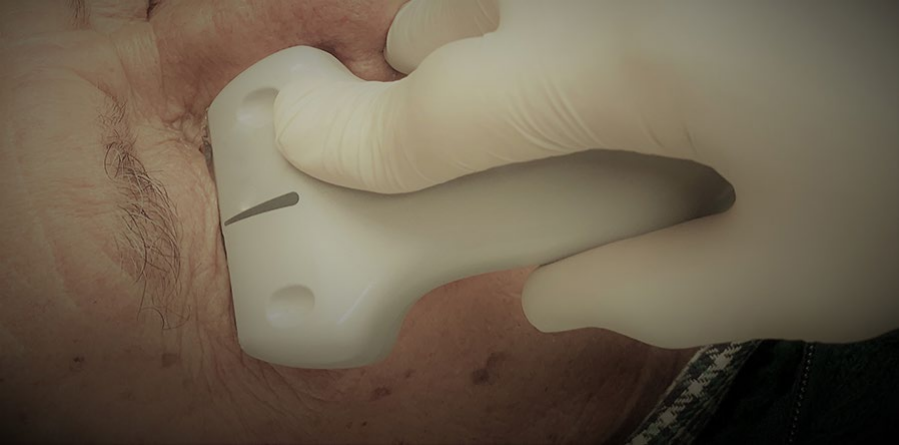
Ultrasonographic pupillary assessment. The 7.5–15 MHz linear transducer gently placed over the lower edge of the closed eye for a trans-palpebral tangential view

The ultrasonographic pupillary assessment included evaluation of bilateral pupillary diameter and pupillary light reflex. With the ultrasound machine on “small parts” preset and the patient in a supine, semi-recumbent position, a linear 7.5–15 MHz transducer was gently placed over the lower edge of the closed eye for a trans-palpebral tangential view, with the probe marker pointed toward the right side of the patient (Fig. 1).

Following FDA regulations for mechanical index and thermal index for ophthalmic ultrasound, we used values less than 0.23 for the mechanical index and less than 1.0 for the thermal index. Although we use the preset small parts, we lowered the mechanical index below 0.23 by changing the acoustic power.

**Fig. 2:**
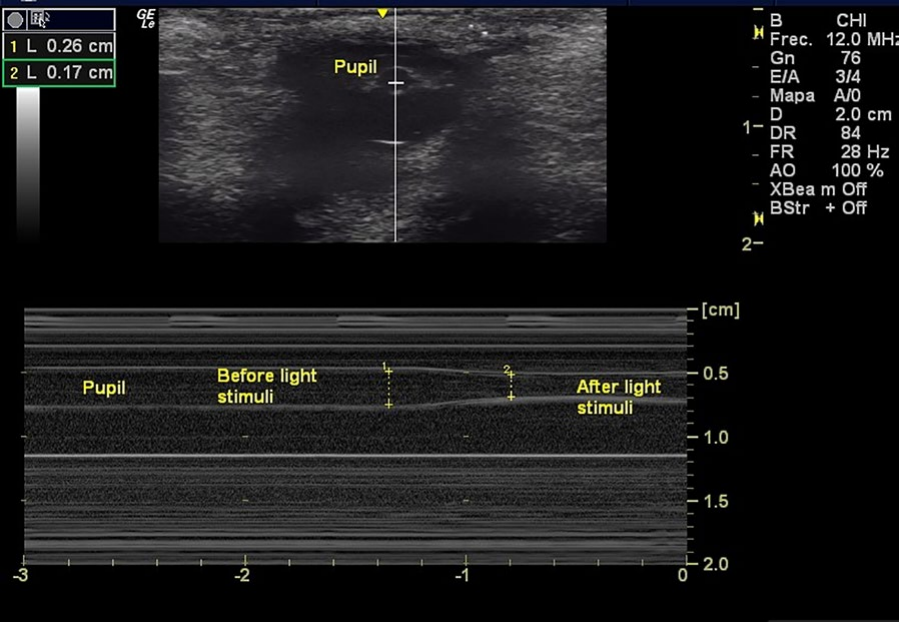
Ocular ultrasound on 2D (top) and M-mode (bottom) showing pupil before light stimuli and after light stimuli. Pupillary on M-mode tracing is highlighted by two white, horizontal, straight, parallel lines

Subsequent tilting movement of the probe was applied until the pupil is visualized. Then, on each eye at a time, over the closed eye and across the eyelid, a light stimulus was shown first ipsilateral to assess for direct pupillary light reflex. Only motion-mode (M-mode) ultrasonographic modality was used for the measurements (Fig. 2). The overall time for UPA per patient, including right and left eye, was never more than 3 min. Although the ultrasound machine is designed to produce minimal risk to the user and the patient, adverse events, such as eye discomfort, irritation, redness or pain, due to superficial pressure exerted by the transducer or due to eyelid and/or eye contact with the sonographic gel, if any, were documented.

### Infrared pupillary assessment (IPA)

**Fig. 3:**
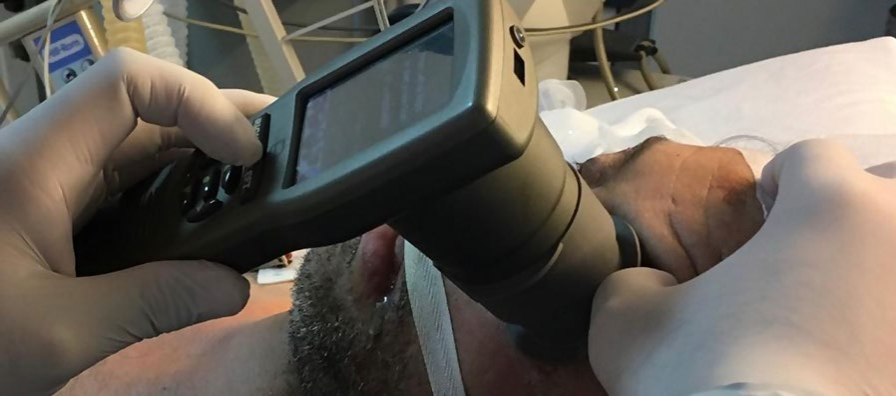
Infrared pupillary assessment. The pupillometer is placed over the open eye, which shows a light stimulus to assess for the pupillary light reflex

The infrared pupillary assessment included evaluation of bilateral pupillary diameter and pupillary light reflex. The infrared pupillometer is a monocular, stand-alone, hand-held, battery-operated instrument, which captures and analyzes the images in less than 3 s. When placed over the eye, the infrared pupillometer shows a light stimulus over the open eye in order to assess for the pupillary light reflex (Fig. 3). The overall time for IPA per patient, including right and left eye, was never more than 1 min. The infrared pupillometer machine is designed to produce minimal risk to the user and the patient. The only designated mechanical contact point with the patient is the headrest. All levels of radiation fall below threshold values recommended by the International Commission on Non-Ionizing Radiation Protection.

For both UPA and IPA, normal values of pupillary diameter were 2 to 4 mm. A pupillary diameter less than 2 mm was documented as miosis, whereas a pupillary diameter greater than 4 mm was documented as mydriasis. The pupillary light reflex was determined by the percentage of reduction in size of pupil diameter immediately after light stimuli. Such a light stimuli was given by the flashlight of a mobile phone model iPhone 6, iPhone 7, iPhone 8 or iPhone X, at an approximate distance of 8 to 12 cm from the eye, and at an angle of 90 degrees (i.e., perpendicular) to the coronal plane of the pupil, all of which have the same documented luminosity, regardless of remaining battery, according to Apple Inc. and were available to the research staff.

The reduction in pupillary diameter was calculated as follows: (maximum resting aperture − minimum aperture)/maximum resting aperture; where maximum resting aperture was the maximal pupillary diameter recorded before light stimuli was shown, minimum aperture was the minimal pupillary diameter recorded after light stimuli was shown. A normal pupillary light reflex was considered as percentage of reduction in size of pupil diameter after light stimuli of 10% or more according to a large study by Taylor et al. [7].

### Statistical analysis

For the comparison between UPA and IPA, Bland–Altman and dispersion plots, simple linear regression models, and the Chi-square test were used. The Shapiro–Wilk test and Q–Q plots were used to corroborate normal distribution of the data. All calculations were done using the R programming language and statistical software (version 3.5.1).

## Results

Total of patients included were 26, most were men (60.0%), with a mean age of 45,05 (range 18–84) years, and mean glasgow coma score at the moment of the pupillary assessment of 8,69 (range 3–15). A total of 212 pupillary measures were obtained, of which 106 were on each eye (i.e., right and left eyes), and 108 were in neurocritically ill patients. Table 1 shows patients classified according to main pathology for ICU admission.

**Table 1:** Absolute frequency represents the number of times the value is present for each variable

Clinical categorization	Absolute frequency (*n*)	Relative frequency (%)
Medical	106	50.0
Medical and surgical	66	31.2
Neurocritical	16	7.5
Surgical	24	11.3
Total	212	100.0

The relative frequency indicates the proportion of the population that correspond to that variable value

Frequencies and percentages of patients according to main pathology for intensive care unit admission.

**Fig. 4:**
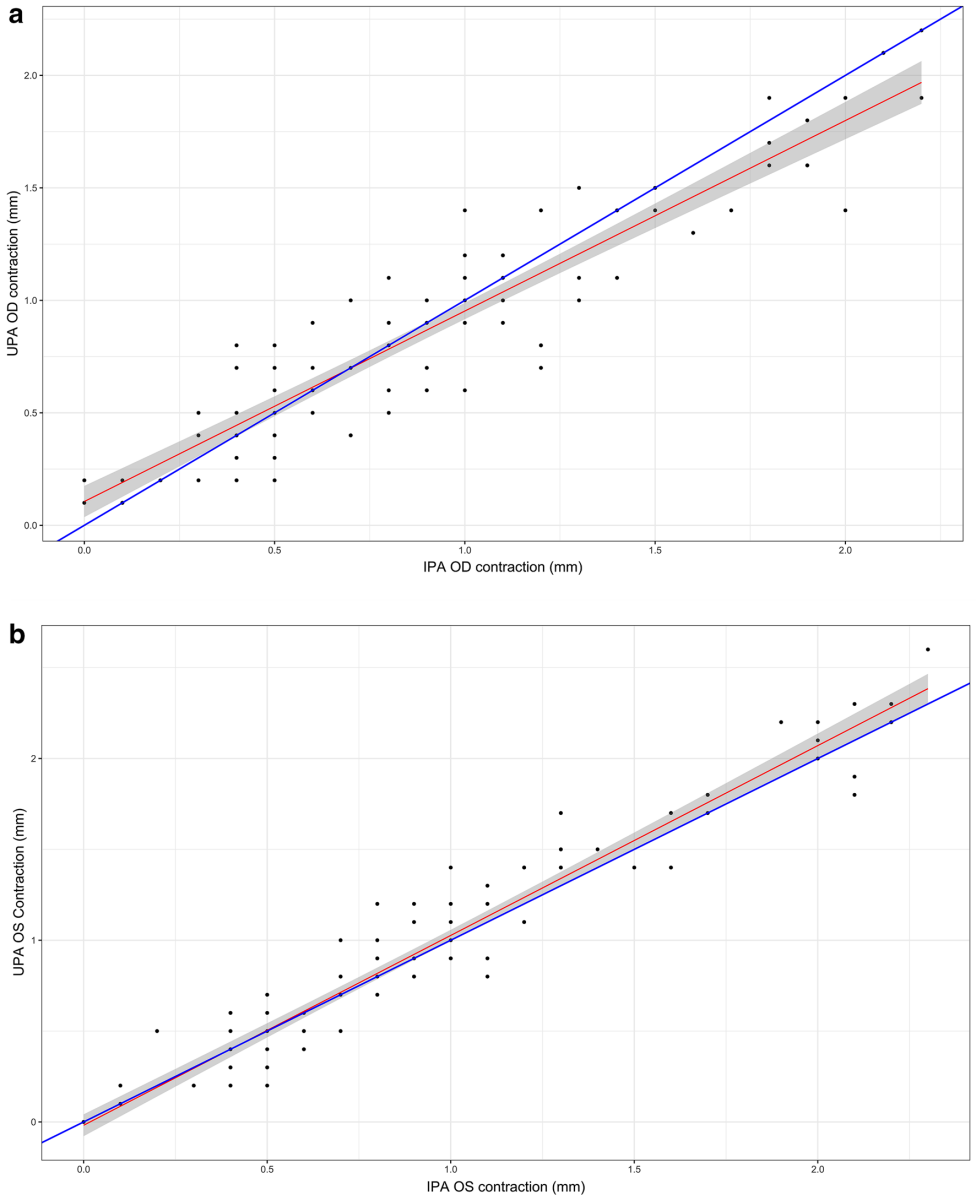

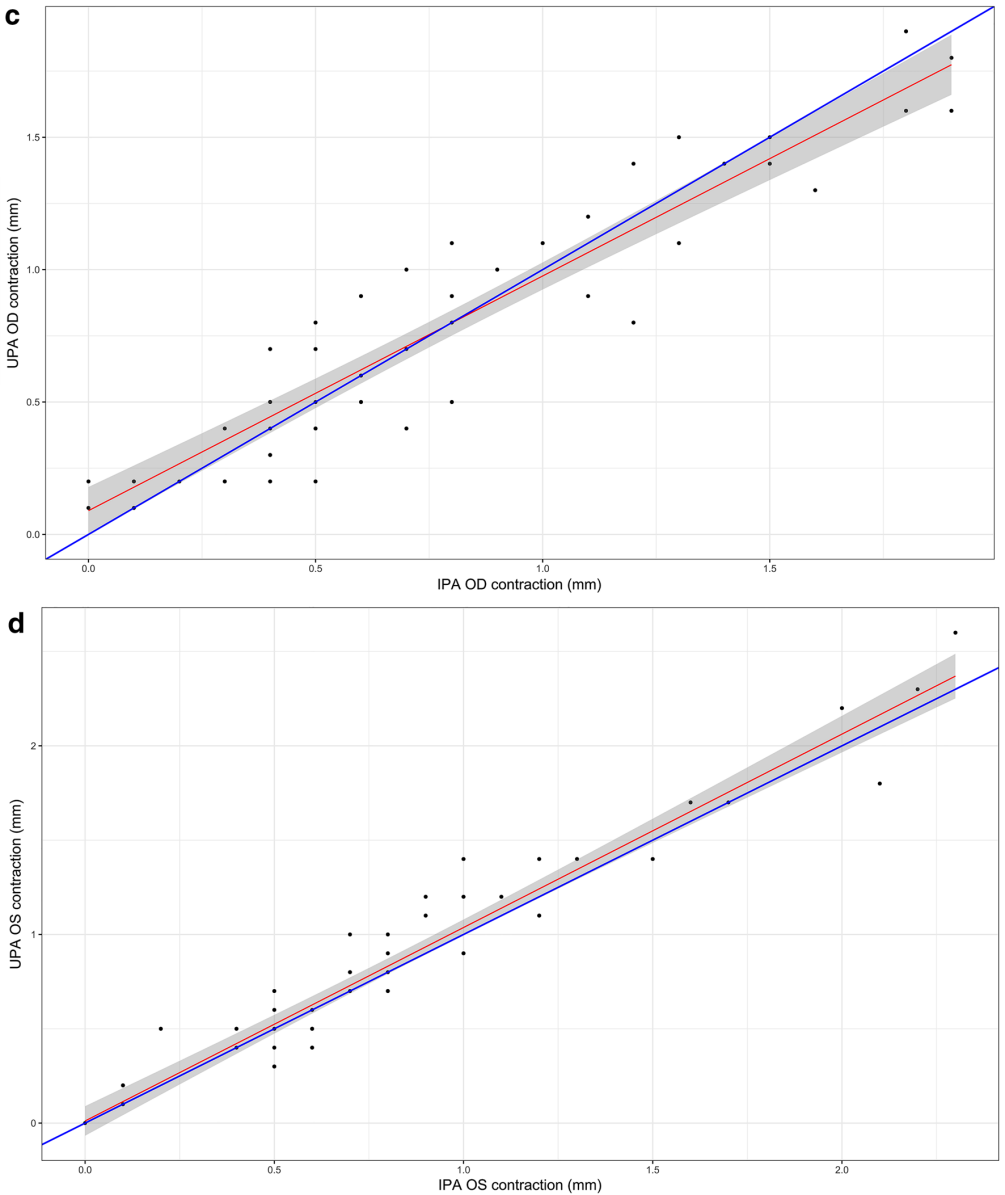
Scatterplot. Panels **a** and **b** demonstrate correlation between UPA and IPA for OD and OS, respectively. Panels **c** and **d** demonstrate correlation between UPA and IPA for OD and OS, respectively, in neurocritically ill patients. The blue line would be the perfect match between both techniques. The red line is the fit of a regression on the set of points

Ultrasonographic pupillary assessment was feasible in all patients. There was a strong positive correlation between measures of reduction in pupillary diameter (pupillary light reflex) obtained by IPA and UPA (r_OD_ = 0.926, 95% CI 0.893–0.949, *p*-value < 0.001; r_OS_ = 0.965, 95% CI 0.949–0.976, *p*-value < 0.001) (Fig. 4a, b).

In the subgroup of patients with critical neurological pathology, a total of 108 measures were obtained with UPA and IPA (54 for OD, 54 for OS) in 8 patients. There was found a strong positive association between UPA and IPA (*r*OD = 0.935, *p* value < 0.001; rOS = 0.965, *p* value < 0.001) (Fig. 4c, d).

**Fig. 5:**
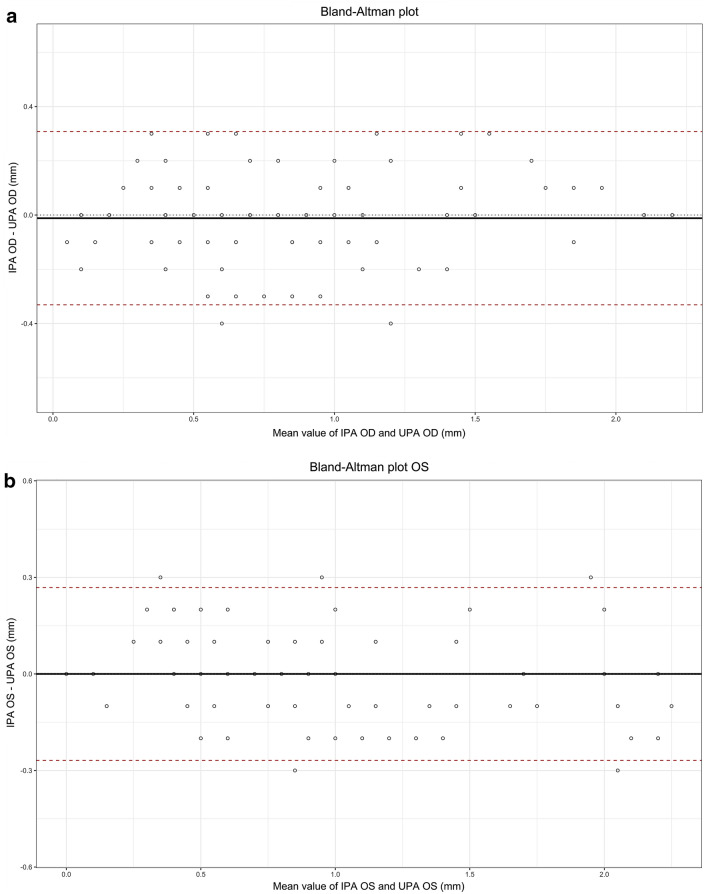

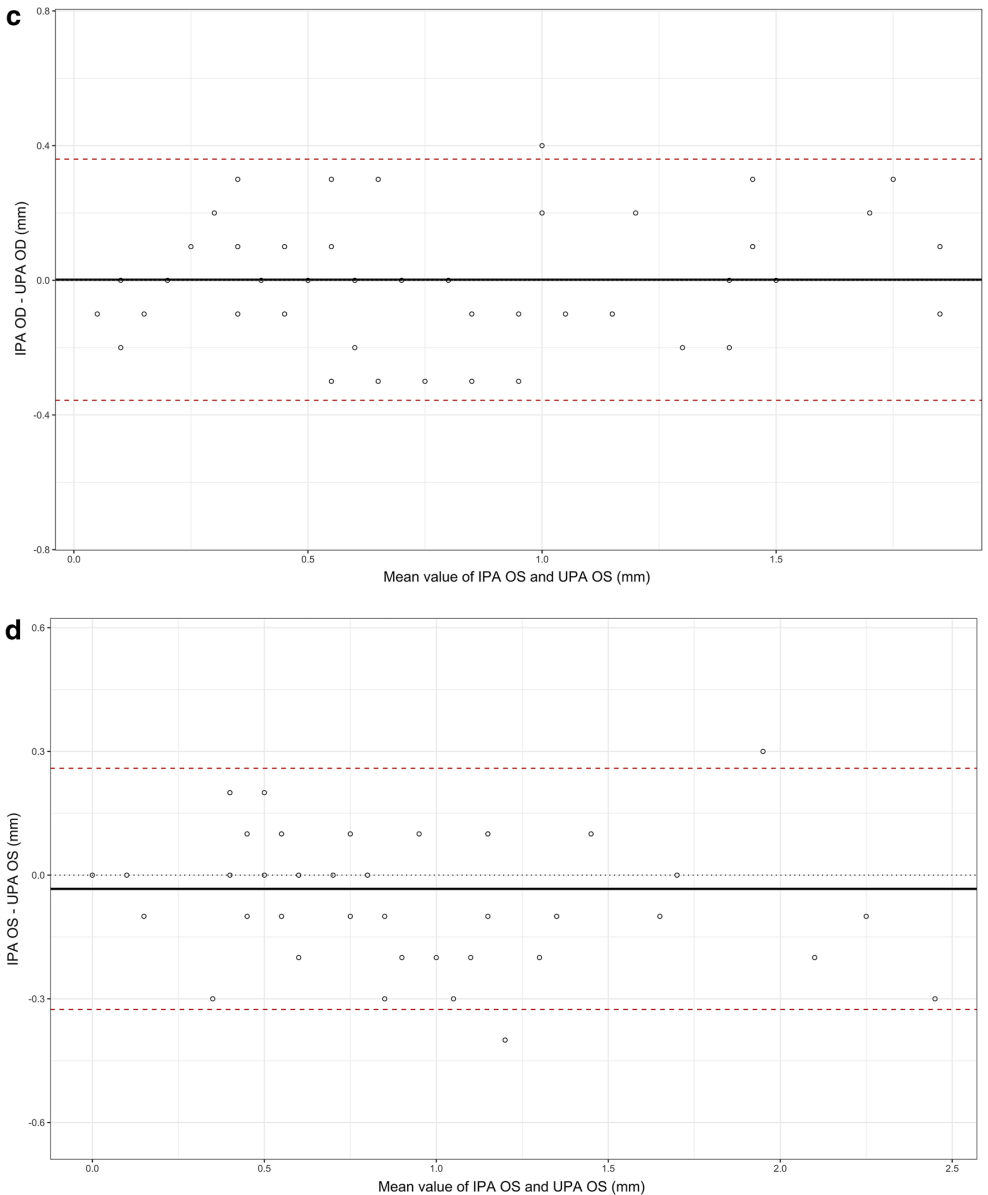
Bland–Altman graphs show the agreement between the IPA and UPA. Panels **a** and **b** demonstrate the agreement between UPA and IPA for OD and OS, respectively. Panels **c** and **d** demonstrate the agreement between UPA and IPA for OD and OS, respectively, in neurocritically ill patients. The solid lines represent the average of the differences; dashed lines indicate limits of agreement (mean ± 1.96 times the standard deviation)

Adjusted analysis through simple linear regression models showed good concordance between IPA and UPA (regression line for OD, *y* = 1, 013*x* + 0, 0192; regression line for OS, *y* = 0.8915*x* + 0.0806). Upon evaluation of normal distribution of the data, 11 measures for OD (differences between-technique measures greater than 0, 3 mm) and 7 for OS (differences between-technique measures less than − 0, 3 mm) were discarded. Taking IPA as reference measure, the percent error for all subjects was 2.77% and 2.15% for OD and OS, respectively (Fig. 5a, b). Regarding concordance between IPA and UPA for measures of reduction in pupillary diameter that were less than 10% (i.e., abnormal), techniques differed in 1 observation for OD and 3 observations for OS, over a total of 106 observations for each eye.

In the subgroup of patients with critical neurological pathology, adjusted analysis through simple linear regression models showed good concordance between IPA and UPA (regression line for OD, *y* = 0, 986*x* + 0.0134; regression line for OS, *y* = 0, 9089*x* + 0, 0483). Taking IPA as reference measure, the percent error for all subjects was 3.21% and 2.44% for OD and OS, respectively (Fig. 5c, d). Regarding concordance between IPA and UPA for measures of reduction in pupillary diameter that were less than 10% (i.e., abnormal), techniques differed in 1 observation for OD and none for OS, over a total of 54 observations for each eye.

In cases of patients with anisocoria (11 observations), defined as a difference of at least 1 mm between resting pupillary diameter, there was also a strong positive association between UPA and IPA (*r* = 0.91, 95% CI 0.68–0.98, *p*-value < 0.001 OD; *r* = 0.954, 95% CI 0.828–0.988, *p*-value < 0.001 OS).

## Discussion

This study found a strong correlation between ultrasonographic and infrared pupillary assessments in critically ill patients, including those with neurologic pathology. UPA could, therefore, be utilized for the evaluation of pupillary diameter and pupillary light reflex in critically ill patients in whom eye opening is not possible. To the authors’ knowledge, this is the first study comparing UPA with IPA.

Unfortunately, the fact that there were very few cases of patients with anisocoria precludes the authors from drawing further conclusions about the role of UPA in this specific patient population. Further studies with a larger patient population are needed.

Regarding the small differences between UPA and IPA, the authors hypothesize that they could be related to the less-intense light stimuli in the case of UPA, because the light stimuli is shown over the closed eye, as opposed to the light stimuli shown directly over the open eye for IPA.

This study shows that UPA is a quick, non-invasive, point-of-care, practical method that provides reliable information, when compared to IPA, regarding pupillary size and pupillary light reflex, which permits assessment of brain herniation. In patients at high risk of brain herniation, UPA becomes particularly useful because it removes the intra- and inter-observer variability inherent to direct visualization of the pupil (i.e., conventional pupillary physical exam). The most important application of UPA would be to provide accurate pupillary assessment in patients in whom eye opening is not possible (e.g., periorbital soft tissue edema) as well as in patients with significant eyelid edema as usually occurs in those with extensive burn injuries. It is not uncommon that patients at risk of or under suspicion of critical neurologic pathology such as brain herniation have concurrent periorbital edema (e.g., traumatic brain injury). UPA could be performed while waiting for computer tomography in the emergency department, ICU, operating room, as well as in emergency pre-hospital settings or underserved areas wherein computed tomography or resonance magnetic imaging is not available.

## Limitations

The small patient population of this study is its major limitation. The greater limitation for UPA is the experience of the operator, for the reliability and accuracy of UPA are directly related to operator skills. Finally, we used different iPhone models that have different technologies generating the flash light used as light stimulus; however, it is unclear whether minor variations in intensity and type of light can significantly affect the assessment of the pupillary light reflex.

## Conclusion

Ultrasonographic pupillary assessment is strongly correlated with infrared pupillary assessment in critically ill patients, including neurocritically ill patients. Ultrasonographic pupillary assessment is a quick, feasible, non-invasive method that allows accurate evaluation of pupillary size and pupillary light reflex in patients in whom a more precise measurement of the pupil is required or eye opening is not possible (e.g., periorbital edema due to traumatic brain injury).

## Data Availability

The datasets used and analysed during the current study are available from the corresponding author on reasonable request.
